# Role of pulsed-xenon ultraviolet light in reducing healthcare-associated infections: a systematic review and meta-analysis

**DOI:** 10.1017/S095026882000148X

**Published:** 2020-07-06

**Authors:** Zhenhong Dong, Na Zhou, Guijuan Liu, Li Zhao

**Affiliations:** 1 Department of Neurosurgery, Zaozhuang Municipal Hospital, Zaozhuang 277101, Shandong Province, P.R. China; 2 Department of Comprehensive Internal Medicine, Zaozhuang Hospital of Traditional Chinese Medicine, Zaozhuang 277100, Shandong Province, P.R. China; 3 Department of Emergency, Zaozhuang Municipal Hospital, Zaozhuang 277101, Shandong Province, P.R. China

**Keywords:** *Clostridium difficile*, disinfection, methicillin-resistant *Staphylococcus aureus*, nosocomial infection, vancomycin-resistant enterococci

## Abstract

Pulsed-xenon-ultraviolet light (PX-UVL) is increasingly used as a supplemental disinfection method in healthcare settings. We undertook a systematic search of the literature through several databases and conducted a meta-analysis to evaluate the efficacy of PX-UVL in reducing healthcare-associated infections. Eleven studies were included in the systematic review and nine in the meta-analysis. Pooled analysis of seven studies with before-after data indicated a statistically significant reduction of *Clostridium difficile* infection (CDI) rates with the use of the PX-UVL (incidence rate ratio (IRR): 0.73, 95% CI 0.57–0.94, *I*^2^ = 72%, *P* = 0.01), and four studies reported a reduction of risk of methicillin-resistant *Staphylococcus aureus* (MRSA) infections (IRR: 0.79, 95% CI 0.64–0.98, *I*^2^ = 35%, *P* = 0.03). However, a further four trials found no significant reduction in vancomycin-resistant enterococci (VRE) infection rates (IRR: 0.80, 95% CI 0.63–1.01, *I*^2^ = 60%, *P* = 0.06). The results for CDI and MRSA proved unstable on sensitivity analysis. Meta-regression analysis did not demonstrate any influence of study duration or intervention duration on CDI rates. We conclude that the use of PX-UVL, in addition to standard disinfection protocols, may help to reduce the incidence of CDI and MRSA but not VRE infection rates. However, the quality of evidence is not high, with unstable results and wide confidence intervals, and further high-quality studies are required to supplement the current evidence.

## Introduction

Healthcare-associated infections (HAIs) are a significant problem contributing to increased mortality, prolonged hospital stay and higher healthcare costs [1]. According to a multistate point prevalence survey in the USA, around 648 000 to 1.7 million hospitalised patients were affected by HAI in a single year [2]. A recent systematic review suggests the prevalence of HAI is 3.12% in mainland China, with rates as high as 26.07% in adult intensive care units (ICUs) [3]. Considering the magnitude of the problem, several studies have examined various methods of reducing the incidence of HAI [4–7]. Notably, an overview of the literature by Harbarth *et al*. [8] concluded that active interventions can reduce HAI from 10% to 70%, depending on the healthcare setting, study design and baseline infection rates.

Contaminated hospital surfaces and medical equipment are important sources of pathogen transmission in any healthcare facility. It is generally acknowledged that organisms such as methicillin-resistant *Staphylococcus aureus* (MRSA), vancomycin-resistant enterococci (VRE), *Pseudomonas* spp., *Acinetobacter* spp. and several viruses can survive for days to weeks on dry inanimate surfaces; spores of *Clostridium difficile* may persist on environmental surfaces for several months [9, 10]. While surface cleaning by chemical germicides is widely used by hospitals, the thoroughness of such cleaning is questionable as studies indicate that fewer than half of hospital surfaces are adequately cleaned by manual methods [11, 12]. To overcome these limitations, ‘no-touch’ disinfection methods using hydrogen peroxide and ultraviolet light (UVL) have been introduced in the past decade [13]. UVL devices use either mercury bulbs emitting continuous radiation (UV-C) of wavelength 200–270 nm or, more recently, xenon gas bulbs which emit radiation in short high-intensity pulses encompassing both UVL (100–280 nm) and visible (380–700 nm) spectra [10, 14]. The latter is known as the pulsed-xenon-UVL system (PX-UVL).

Several studies have shown that both UV-C and PX-UVL systems are effective at reducing surface contamination [15–17]. A recent meta-analysis of 13 UVL studies by Marra *et al*. [13] has demonstrated that the introduction of UVL disinfection may help reduce the incidence of *C. difficile* infection (CDI) and VRE infections in healthcare settings. The review, however, had several limitations, the foremost being that studies utilising both UV-C and PX-UVL were combined for the analysis. Nerandzic *et al*. [14] have shown that PX-UV may be less effective than UV-C in reducing MRSA, VRE and *C. difficile* spore count on similar surfaces when used for the same time at an equal distance. Similarly, errors of data entry and combining studies with duplicate data may also have led to inaccuracies in the analysis. As a consequence, results of individual studies on the role of PX-UVL in reducing HAI rates are conflicting. While Levin *et al*. [18] found a significant reduction of HAI rates after the introduction of PX-UVL, no such effect was noted by a recent study of Attia *et al*. [19]. In the absence of any other pooled evidence, it is not known if PX-UVL systems can contribute to a reduction in HAI. Therefore, the purpose of our study was to systematically search the literature for studies assessing the efficacy of PX-UVL in reducing HAI and conduct an accurate meta-analysis to provide high-level evidence.

## Materials and methods

### Search strategy

The PRISMA guidelines (Preferred Reporting Items for Systematic Reviews and Meta-analyses) [20], except for protocol registration, were followed for this systematic review. We searched computer databases such as PubMed, Embase, Scopus, BioMed Central and Cochrane library up to 2 February 2020. The detailed search strategy used for the PubMed database is presented in Supplementary content 1. The search was conducted by two reviewers independently. Literature results were screened by titles and abstracts, and full texts of relevant articles were extracted. Both reviewers assessed individual articles based on the inclusion and exclusion criteria, and any disagreements were resolved by discussion. Post-screening, the bibliography of included studies, as well as review articles on the subject were searched manually for any missed articles.

### Inclusion criteria

We included studies that met the following criteria: (1) they were conducted in any health-care setting (academic, community, tertiary care hospitals, etc.) and (2) had assessed the efficacy of PX-UVL for the reduction of the incidence of HAI. Studies were included irrespective of sample size and language of publication. No restriction was placed on the type of patients included or the site of intervention. HAI was defined as per the included study and no condition was placed on the inclusion of specific infections. Randomised controlled trials (RCTs), non-randomised trials, controlled and uncontrolled before-after studies were included. We excluded studies assessing the effect of other UVL devices on HAI, and those evaluating the efficacy of PX-UVL combined with other infection control measures were also excluded. We did not include studies analysing the effect of PX-UVL on reducing surface contamination. In cases of publications with duplicate data, the study with the largest database was selected.

### Data extraction and risk of bias

Two reviewers extracted data from the included studies independently. Data regarding authors, publication year, study type, its duration and location, intervention site, baseline disinfection methods, the protocol of PX-UVL, study outcomes and study results were extracted. The outcome of interest was a reduction in the incidence of HAI with the use of PX-UVL. The RoBANS tool was used to assess the risk of bias for non-randomised studies [21]. The criteria for assessing the risk of bias included: patient selection, confounding factors, measurement of exposure, blinding of outcome assessment, incomplete outcome data and selective reporting.

### Statistical analysis

‘Review Manager’ (RevMan, version 5.3; Nordic Cochrane Centre (Cochrane Collaboration), Copenhagen, Denmark; 2014) was used for the meta-analysis. HAI data were presented as incidence rates in the included studies. Incidence rate ratios (IRR) were calculated with 95% confidence intervals (CIs) using the ‘fmsb’ package of statistical software R (V.3.5.1) (The R Foundation for Statistical Computing, Vienna, Austria). Data on the number of HAI cases and total person-days were extracted for the calculation of IRR. Study estimates were then combined using inverse variance-weighted averages of logarithmic IRRs in a random-effects model. We conducted a meta-analysis only where at least three studies reported data for the same outcome. Heterogeneity in the analysis was assessed using the *I*^2^ statistic. *I*^2^ values of 25–50% represented low, 50–75% medium and >75% *r* substantial heterogeneity. *P*-values of <0.05 were considered statistically significant. A sensitivity analysis was carried out to assess the influence of each study on the pooled effect size. Due to the inclusion of fewer than 10 studies in the meta-analysis, funnel plots were not used to assess publication bias. A random-model meta-regression analysis was performed for meta-analyses including more than five studies using meta-essentials [22]. The influence of the duration of the study and the duration of the intervention period on the log-transformed values of IRR was assessed.

## Results

### Search results and study characteristics

**Fig. 1.**
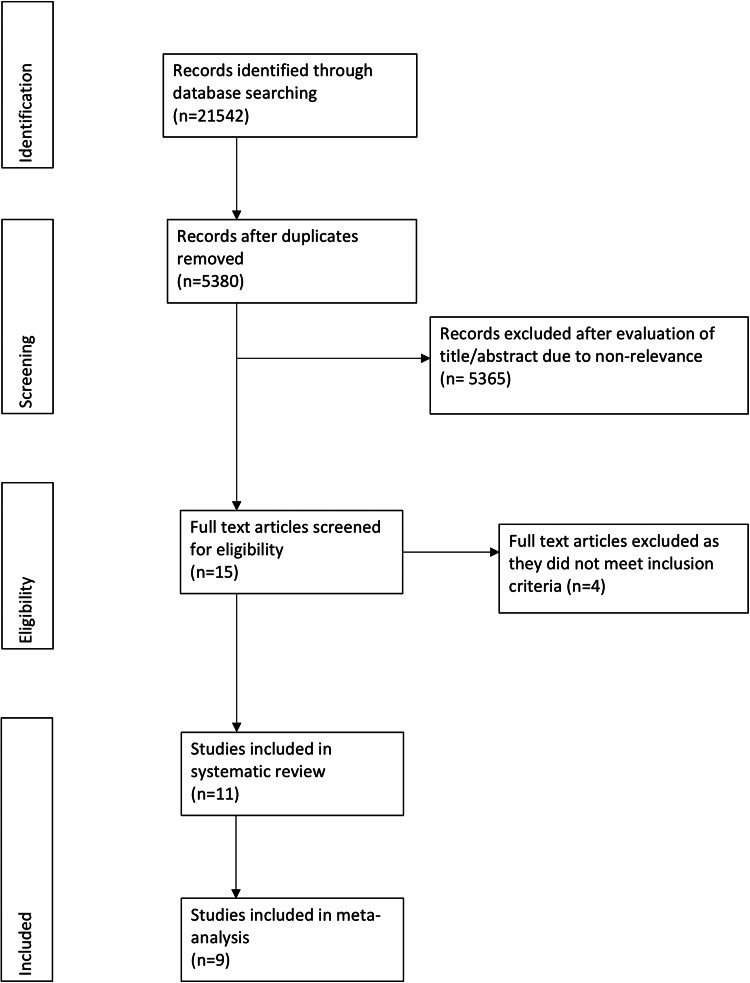
PRISMA flow chart of the study.

A PRISMA flowchart of the study is presented in Figure 1. Fifteen articles were selected for full-text analysis after the literature search. Four studies were excluded as, two did not utilise PX-UVL systems [23, 24], one reported overlapping data [25] with an included study [26] and the other assessed the combination of screening, hand hygiene education and PX-UVL on HAI rates [27]. A total of 11 studies were included in the systematic review [18, 19, 26, 28–35].

Baseline details of the included studies are presented in Table 1. Except for a recent study in Japan [28], all trials were conducted in the USA; 10 were uncontrolled before-after studies and one was a controlled clinical trial [29]. However, to maintain homogeneity, before-after data of only the intervention arm were extracted for the meta-analysis from this study. The duration of studies varied from 18 to 52 months and were conducted in different healthcare facilities including tertiary care [19, 26, 28, 29, 31], community [18, 30, 32], long-term care hospitals [33, 34] and a burn centre [35]. The intervention site varied amongst studies with use of PX-UVL systems in all patient rooms [18, 33–35], operating rooms [18, 26, 32, 35], ICUs [19, 28, 30], contact precaution rooms [26, 30], haematological or bone marrow transplant (BMT) units [19, 29, 31], paediatric units [19], medical–surgical units [19, 29], dialysis unit [26] and burn units [26, 35]. Baseline disinfection protocols were reported to be similar during the pre-intervention and post-intervention period in all trials. PX-UVL was utilised after baseline cleaning in all studies; the majority used 5-min cycles of PX-UVL for disinfection of hospital rooms and the number of cycles varied from 2 to 4. Except for three studies [19, 31, 35], all trials reported a significant reduction in HAI rates after the use of PX-UVL in their establishment.

**Table 1.** Characteristics of included studies

Author/Year	Country	Study type	Duration of study (months)	Study location	Intervention site	Standard disinfection method	Protocol of PX-UVL use	Study outcomes	Study results
Levin *et al*./2013 [18]	USA	Before-after	24	140-bedded acute care community hospital	All patient rooms, operating rooms, emergency department	Cleaning by hospital grade disinfection product in all rooms. Chlorhexidine wipes used in CD rooms	Used after terminal cleaning, for three 7-min cycles (2 in patient room, 1 in bathroom)	CDI	Significant reduction of HAI-CD rates, deaths and colectomy with use of PX-UVL
Haas *et al*./2014 [26]	USA	Before-after	52	643-bedded tertiary care hospital	Contact precautions room, operating rooms, dialysis unit and burn unit	Cleaning by sodium hypochlorite and quaternary ammonium based-compound	Used after terminal cleaning, for three 6-min cycles (2 in patient room, 1 in bathroom). Used daily in operating rooms, weekly in dialysis and burn unit	CDI, HAI by MDRO (VRE, MRSA, Gram-negative bacteria)	Significant reduction of HAI-MDRO and CD rates with use of PX-UVL
Miller *et al*. [34]	USA	Before-after	38	Long-term acute-care hospital	All patient rooms and communal living areas	Cleaning by sodium hypochlorite	Used after terminal cleaning as an adjunct to standard cleaning practices	CDI	Significant reduction of HAI-CD rates with use of PX-UVL
Catalanotti *et al*./2016 [32]	USA	Before-after	36	200-bedded community hospital	13 operating rooms	Nightly cleaning and between-case cleaning with unreported methods	Used after terminal cleaning by a dedicated staff using 2 PX-UVL systems simultaneously for 10-min cycle	Surgical site infection rates	Significant reduction of surgical site infection for clean but not clean-contaminated procedures
Vianna *et al*./2016 [30]	USA	Before-after	44	206-bedded community hospital	ICU and contact precautions room	Standard cleaning using bleach	Used after terminal cleaning for three 5-min cycles (2 in patient room, 1 in bathroom)	CDI, HAI by MDRO (VRE, MRSA)	Significant reduction of HAI-CD rates in non-ICUs. In the ICU, all infections were reduced, but only VRE was significant
Green *et al.*/2017 [35]	USA	Before-after	18	Burn centre	Patient rooms, operating rooms, shower rooms, ancillary areas	Cleaning with hospital-approved disinfectants	Used after terminal cleaning or when room vacated for procedure for four 5-min cycles in patient rooms and two cycles for shower rooms, ancillary areas (daily). Used for two cycles of 10-min in operating rooms	CDI, HAI by MDRO (MDR Gram-negative bacteria, MRSA), CLABSI, CAUTI, VAP	No statistical significant impact on device-associated infection rates and HAI-MDRO
Kovach *et al*./2017 [33]	USA	Before-after	48	160-bedded long-term care facility	All patient rooms	Cleaning with sodium hypochlorite and detergent cleaning solution	Used after terminal cleaning for three 5-min cycles (2 in patient room, 1 in bathroom)	All HAI	Significant reduction in HAI and hospitalisations for infection
Brite *et al*./2018 [31]	USA	Before-after	21	474-bedded tertiary care facility	25-bedded BMT unit	Cleaning by sodium hypochlorite and quaternary ammonium based-compound	Used after terminal cleaning for three 5-min cycles (2 in patient room, 1 in bathroom) for terminal disinfection. One 5-min cycle daily for bathrooms	CDI, HAI by VRE	No statistical significant reduction of HAI-CD and VRE rates
Sampathkumar *et al*./2019 [29]	USA	Controlled clinical trial	27	2059-bedded tertiary care facility	2 haematological and BMT units and 1 medical–surgical unit each for intervention and control	Bleach used for all haematological and BMT units and rooms of medical surgical units with known CD infection	Used after terminal cleaning for three 5-min cycles	CDI, HAI by VRE	Significant reduction of HAI-CD and VRE rates with use of PX-UVL
Attia *et al*./2020 [19]	USA	Before-after	18	500-bedded academic tertiary care facility	Surgical ICU, medical ICU, medical intermediate care unit, adult haematology-oncology unit, paediatric haematology-oncology unit, paediatric acute unit	Cleaning using sodium hypochlorite/hydrogen peroxide	Used after terminal cleaning for three 5-min cycles (2 in patient room, 1 in bathroom)	CDI	No statistical significant reduction of HAI-CD rates
Morikane *et al.*/2020 [28]	Japan	Before-after	30	629-bedded tertiary academic referral hospital	ICU with 6 rooms and beds	Cleaning with sodium hypochlorite solution	Used after terminal cleaning for two 5-min cycles	HAI-MRSA and 2-drug-resistant *Acinetobacter baumannii*	Significant reduction of HAI-MRSA and 2-drug-resistant *A. baumannii*

PX-UVL, pulsed-xenon-ultraviolet light; ICU, intensive care unit; HAI, Healthcare-associated infection; CDI, *Clostridium difficile* infection; MDRO, multidrug-resistant organism; VRE, vancomycin-resistant *enterococci*; MRSA, methicillin-resistant *Staphylococcus aureus*; MDR, multi-drug-resistant; CLABSI, central line associated bloodstream infection; CAUTI, catheter associated urinary tract infection; VAP, ventilator associated pneumonia; BMT, bone marrow transplant

### Outcomes

While all studies compared the incidence of HAI before and after the introduction of PX-UVL, there was variation in the type of HAI analysed. Based on the availability of data, a meta-analysis was conducted for healthcare-associated CDI, MRSA and VRE infections. Catalanotti *et al*. [32] in their study of operating rooms reported the incidence of surgical site infections only while Kovach *et al*. [33] cited cumulative HAI rates in a 160-bed long-term care facility, irrespective of the organism. Although both reported a significant reduction in infection rates, these studies were not included in the meta-analysis.

### Analysis

**Fig. 2.**
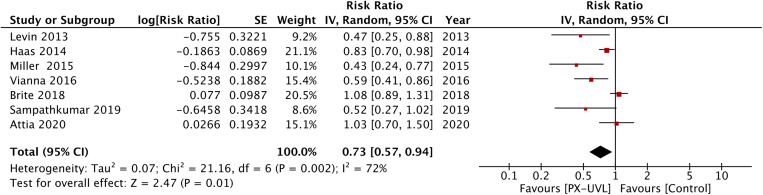
Forest plot of IRRs of CDI for PX-UVL *vs*. control.

**Fig. 3.**
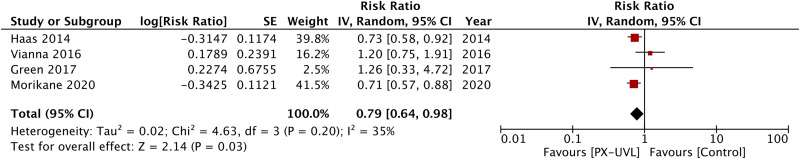
Forest plot of IRRs of MRSA infection for PX-UVL *vs*. control.

**Fig. 4.**
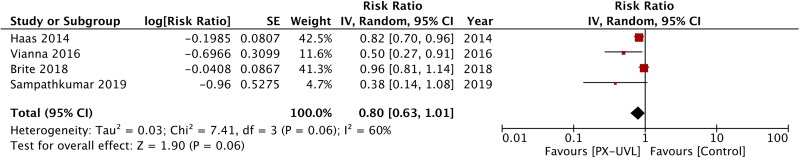
Forest plot of IRRs of VRE infection for PX-UVL *vs*. control.

A total of seven studies reported data on CDI rates [18, 19, 26, 29–31, 34]. Pooled analysis indicated a significant reduction of CDI rates with the use of PX-UVL (IRR: 0.73, 95% CI 0.57–0.94, *I*^2^ = 72%, *P* = 0.01) (Fig. 2). On the pooling of data from four studies on MRSA infection rates [26, 28, 30, 35], our analysis indicated that PX-UVL reduces the risk of healthcare-associated MRSA infections (IRR: 0.79, 95% CI 0.64–0.98, *I*^2^ = 35%, *P* = 0.03) (Fig. 3). In contrast, a similar pool of four trials [26, 29–31] revealed no significant reduction in VRE infection rates (IRR: 0.80, 95% CI 0.63–1.01, *I*^2^ = 60%, *P* = 0.06) (Fig. 4).

**Fig. 5.**
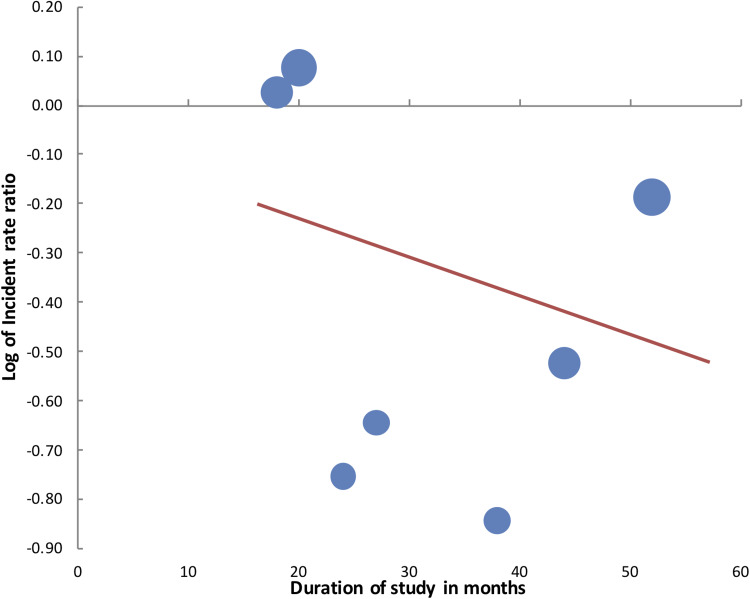
Meta-regression plot for influence of moderator ‘study duration’ on log of IRRs of CDI.

**Fig. 6.**
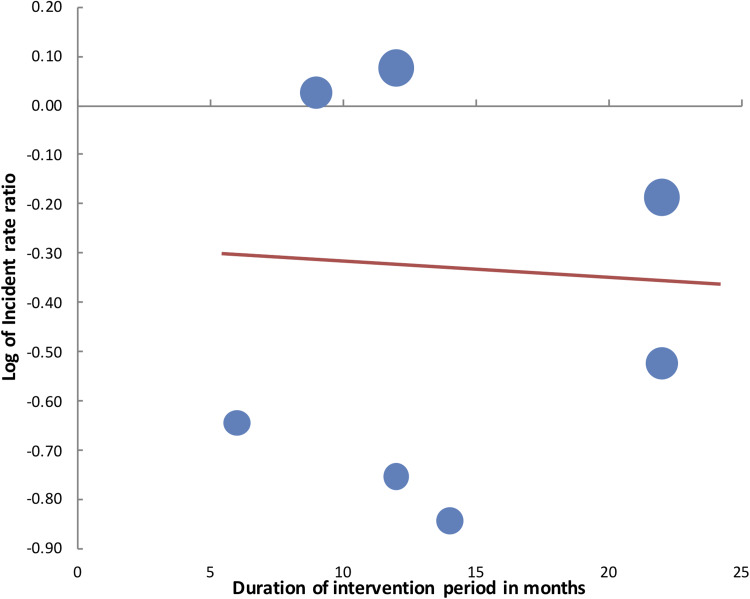
Meta-regression plot for influence of moderator ‘intervention duration’ on log of IRRs of CDI.

The sensitivity analysis is shown in Table 2. For CDI, the results became statistically non-significant on the exclusion of the studies of Miller *et al*. [34] and Vianna *et al*. [30]. Similarly, exclusion of the studies of Haas *et al*. [26] and Morikane *et al*. [28] from MRSA analysis, resulted in a change in the significance of effect size which indicated no benefit of PX-UVL in reducing MRSA infection rates. VRE infection rates were stable on sensitivity analysis. Meta-regression analysis for the moderator ‘duration of study’ on CDI did not demonstrate any significant influence on the effect size (*β*:−0.01, 95% CI −0.03 to 0.02, *P* = 0.48) (Fig. 5). Similarly, no significant influence was seen on the ‘duration of intervention period’ on CDI rates (*β*:−0.003, 95% CI −0.065 to 0.058, *P* = 0.89) (Fig. 6).

**Table 2.** Results of sensitivity analysis on sequential exclusion of each study

Outcome	Excluded study	Resultant effect size
HAI-*Clostridium difficile*	Levin *et al*. [18]	IRR: 0.77, 95% CI 0.60–0.99, *I*^2^ = 72%, *P* = 0.04
	Haas *et al*. [26]	IRR: 0.68, 95% CI 0.48–0.97, *I*^2^ = 76%, *P* = 0.03
	Miller *et al*. [34]	**IRR: 0.79, 95% CI 0.62–1.00, *I*^2^ = 68%,** *P* **= 0.05**
	Vianna *et al*. [30]	**IRR: 0.76, 95% CI 0.59–1.00, *I*^2^ = 71%,** *P* **= 0.05**
	Brite *et al*. [31]	IRR: 0.67, 95% CI 0.51–0.87, *I*^2^ = 59%, *P* = 0.003
	Sampathkumar *et al*. [29]	IRR: 0.76, 95% CI 0.59–0.98, *I*^2^ = 74%, *P* = 0.003
	Attia *et al*. [19]	IRR: 0.68, 95% CI 0.52–0.91, *I*^2^ = 75%, *P* = 0.008
HAI-MRSA	Haas *et al*. [26]	**IRR: 0.90, 95% CI 0.59–1.38, *I*^2^ = 54%,** *P* **= 0.64**
	Vianna *et al*. [30]	IRR: 0.73, 95% CI 0.62–0.85, *I*^2^ = 0%, *P* < 0.0001
	Green *et al*. [35]	IRR: 0.79, 95% CI 0.63–0.99, *I*^2^ = 51%, *P* = 0.04
	Morikane *et al*. [28]	**IRR: 0.91, 95% CI 0.61–1.35, *I*^2^ = 48%,** *P* **= 0.63**
HAI-VRE	Haas *et al*. [26]	IRR: 0.86, 95% CI 0.70–1.05, *I*^2^ = 53%, *P* = 0.14
	Vianna *et al*. [30]	IRR: 0.65, 95% CI 0.36–1.17, *I*^2^ = 71%, *P* = 0.15
	Brite *et al*. [31]	IRR: 0.64, 95% CI 0.40–1.00, *I*^2^ = 53%, *P* = 0.05
	Sampathkumar *et al*. [29]	IRR: 0.84, 95% CI 0.67–1.03, *I*^2^ = 60%, *P* = 0.10

IRR, incidence rate ratio; MDRO, multidrug-resistant; MRSA, methicillin-resistant *Staphylococcus aureus*; VRE, vancomycin-resistant *enterococci*.

Results with change in significance of effect size from the primary analysis are highlighted in bold.

### Risk of bias

The risk of bias assessment of included studies is presented in Table 3. All studies included a similar patient population in the same setting. Confounding factors such as hand hygiene compliance and efficiency of baseline cleaning was reported only by Brite *et al*. [31]. Due to the study design, none of the trials was blinded. Less than 90% compliance with PX-UVL systems was reported by four studies [18, 26, 29, 31]. Since none of the included studies had a pre-defined protocol, selective reporting could not be evaluated.

**Table 3.** Risk of bias in included studies

Author	Selection of participants	Confounding variables	Measurement of exposure	Blinding of outcome assessment	Incomplete outcome data	Selective outcome reporting
Levin *et al.* [18]	Low risk	High risk	Low risk	High risk	High risk	Unclear risk
Haas *et al*. [26]	Low risk	High risk	Low risk	High risk	High risk	Unclear risk
Miller *et al*. [34]	Low risk	Unclear risk	Low risk	High risk	Unclear risk	Unclear risk
Catalanotti *et al*. [32]	Low risk	High risk	Low risk	High risk	Unclear risk	Unclear risk
Vianna *et al*. [30]	Low risk	High risk	Low risk	High risk	Unclear risk	Unclear risk
Green *et al*. [35]	Low risk	High risk	Low risk	High risk	Unclear risk	Unclear risk
Kovach *et al.* [33]	Low risk	High risk	Low risk	High risk	Low risk	Unclear risk
Brite *et al.* [31]	Low risk	Low risk	Low risk	High risk	High risk	Unclear risk
Sampathkumar *et al*. [29]	Low risk	Unclear risk	Low risk	High risk	High risk	Unclear risk
Attia *et al*. [19]	Low risk	High risk	Low risk	High risk	Unclear risk	Unclear risk
Morikane *et al.* [28]	Low risk	High risk	Low risk	High risk	Unclear risk	Unclear risk

## Discussion

To augment manual cleaning of hospital surfaces, ‘no-touch’ systems like PX-UVL have been developed to reduce the incidence of HAI. Our meta-analysis indicates that the use of PX-UVL may reduce the incidence of healthcare-associated CDI and MRSA infections but has no demonstrable effect in reducing VRE infection rates.

The PX-UVL device has been marketed as an efficient germicidal appliance capable of significantly reducing surface contamination with pathogens. A high-intensity UVL is delivered in millisecond pulses which is capable of damaging DNA, RNA and proteins of bacteria, viruses and spores. Four mechanisms are suggested for its action namely, photohydration (pulling water molecules into the DNA), photo-splitting (breaking the DNA), photodimerisation (improper fusing of DNA bases) which prevents cell replication; and photocrosslinking which causes irreversible cell wall damage and cell death [33]. UV-C devices also act by inducing DNA and RNA damage to prevent microbial replication [36]. There are, however, some differences between the two devices. Since PX-UVL does not contain mercury, safety hazards related to mercury exposure are reduced. Similarly, the manufacturer recommended disinfection cycle is shorter for PX-UVL as compared to UV-C devices (10–20 *vs.* 45 min) [14]. Organic matter does not appear to influence the penetration of PX-UVL [37], but the efficiency of UV-C for the killing of spores is modestly reduced in the presence of organic matter [37]. On the other hand, UV-C may be more efficient in reducing pathogens on glass slides than PX-UVL [14]. In the absence of comparative studies of PX-UVL *vs.* UV-C in real-world clinical scenarios, it is not known if such differences have any effect on HAI rates.

Owing to these documented dissimilarities between the two systems, this review was focused exclusively on evaluating the efficacy of PX-UVL for reducing HAI. Our analysis indicated a 27% reduced risk of CDI when PX-UVL was supplemented with standard cleaning. However, the CIs of the calculated risk ratio were wide-ranging from 6% to 43%; also, results were not stable on sensitivity analysis after the exclusion of two studies which individually resulted in a change in statistical significance of the result. The previous meta-analysis of a combination of UV-C and PX-UVL also demonstrated a 36% (95% CI 16–51) reduced risk of CDI infections [13]. However, on closer inspection, the latter had included the study of Nagaraja *et al*. [25] which is a further analysis of an already included trial of Haas *et al*. [26]. Moreover, there were inaccuracies in calculating the IRR of several studies in their analysis which resulted in wide CIs. For example, the calculated IRR of Vianna *et al*. [30] in the analysis was 0.59% (95% CI 0.02–20.25) when the correct calculation based on the number of cases and the total number of person-days is 0.59 (95% CI 0.41–0.86). We believe the IRR was calculated in their study based on the incidence rate per 1000 days which resulted in the said errors.

PX-UVL was found to significantly reduce the risk of MRSA but not of VRE. Meta-analysis indicated a 21% reduced risk of MRSA infections with the use of PX-UVL, albeit with a wide CI (2–36%). These results were also unstable on sensitivity analysis with exclusion of two of the four included studies resulting in no statistical difference. The inconsistency and instability of our results may partly be explained by the limited number of studies available for analysis. Also, the results of the 11 studies were conflicting with three trials [19, 31, 35] reporting no significant difference in HAI rates. Since the latter studies were of shorter duration (18–21 months), a meta-regression analysis was conducted to assess the influence of study duration and intervention arm duration on the effect size; no impact of these moderators was evident for CDI.

It is important to note that all our results are based on data of before-after studies. To date, only one RCT [24] has evaluated the efficacy of UVL for reducing HAI. This was a multi-centric crossover trial in nine hospitals in the USA that evaluated the role of a UV-C device in reducing MRSA, VRE and CDI. While there was a significant reduction of VRE infections with the combined use of bleach and UV-C, no difference was noted for MRSA and CDI when this combination was compared to bleach cleaning alone. The lack of additional effectiveness of UV-C in their study was attributed to higher compliance of baseline bleach cleaning (90%) and the use of a single cycle of UV-C placed adjacent to, but outside the bathroom. In the only controlled trial of PX-UVL, Sampathkumar *et al*. [29] reported a statistically significant reduction of CDI and VRE infection rates in their hospital units utilising PX-UVL compared with similar units not employing the disinfection system. The machines of their study were donated by the equipment manufacturer.

There are certain disadvantages of PX-UVL systems which may hinder widespread use in healthcare settings. Foremost, they are expensive and require additional training and manpower for routine use [29]. Also, the appliance emits an intense light and sound when in use which may be unacceptable to patients and healthcare workers. It is therefore recommended to be used in empty rooms [28]. This may be feasible in hospitals employing private rooms for all patients but is unlikely to be practicable in lower-income countries where hospital rooms are usually shared. Use of the device after standard disinfection protocols increases the total time of disinfection per room, however, reports suggest it to be insignificant [28, 29]. The disinfection efficacy of PX-UVL is dramatically reduced as the distance from the device increases [14] and high-touch surfaces need to be brought closer to the apparatus for optimal disinfection. There is also a potential negative impact of shadows, room size and configuration on the disinfection efficiency [19].

The limitations of our review need to be considered. First, none of the included studies were RCTs and only before-after data were analysed. A comparison with a historical control group does not take into account changes in hospital practices over time. Second, there were variations in the study locations, intervention site and baseline standard disinfection methods employed in the included studies. The incidence of HAI can vary in different hospital sites with generally a higher incidence in ICUs and operating rooms compared with single-patient rooms [3]. The varied patient population in the included studies can also influence HAI rates. Burns and cancer patients have reduced immunity and are more prone to HAI compared to patients without comorbidities [31, 35]. Third, many other factors impact on HAI rates such as the use of active surveillance, hand hygiene compliance, efficiency of standard disinfection protocols and compliance with the PX-UVL protocol, among others. Finally, the majority of the studies in our analysis were conducted in the USA; differences in healthcare systems and protocols between the USA and other countries could therefore limit the applicability of our findings worldwide. Nevertheless, our study is the first meta-analysis which exclusively assesses the efficacy of PX-UVL in reducing HAI. In comparison with a previous study [13], we were able to exclude duplicate datasets and add four new studies which served to provide an accurate and more comprehensive review.

Our study indicates that the use of PX-UVL in addition to standard disinfection protocols may help reduce the incidence of CDI and MRSA, but not VRE infection rates. The quality of evidence is not high and compromised by unstable results and wide CIs. Further high-quality studies are required to supplement current evidence.

## Data Availability

The datasets used and/or analysed during the current study are available from the corresponding author on reasonable request.

## Supplementary material

For supplementary material accompanying this paper visit http://dx.doi.org/10.1017/S095026882000148X. click here to view supplementary material
