# Supplementary material for: Role of pulsed-xenon ultraviolet light in reducing healthcare-associated infections: a systematic review and meta-analysis
Source: Epidemiol Infect. 2020 Jul 6;148:e165. doi: 10.1017/S095026882000148X (PMC7424602; doi:10.1017/S095026882000148X)
Supplement: Supplementary file 1 [file S095026882000148Xsup001.docx]

Supplementary content 1: Search strategy and results of PubMed database

| **Search number** | **Query** | **Search Details** | **Results** |
| --- | --- | --- | --- |
| 1 | pulsed xenon ultraviolet light | (((((((("pulse"[MeSH Terms] OR "pulse"[All Fields]) OR "heart rate"[MeSH Terms]) OR ("heart"[All Fields] AND "rate"[All Fields])) OR "heart rate"[All Fields]) OR "pulses"[All Fields]) OR "pulse s"[All Fields]) OR "pulsed"[All Fields]) OR "pulsing"[All Fields]) AND ((("xenon"[MeSH Terms] OR "xenon"[All Fields]) OR "xenon s"[All Fields]) OR "xenons"[All Fields]) AND (((("ultraviolet rays"[MeSH Terms] OR ("ultraviolet"[All Fields] AND "rays"[All Fields])) OR "ultraviolet rays"[All Fields]) OR ("ultraviolet"[All Fields] AND "light"[All Fields])) OR "ultraviolet light"[All Fields]) | 67 |
| 2 | (ultraviolet light) AND (disinfection) | (((("ultraviolet rays"[MeSH Terms] OR ("ultraviolet"[All Fields] AND "rays"[All Fields])) OR "ultraviolet rays"[All Fields]) OR ("ultraviolet"[All Fields] AND "light"[All Fields])) OR "ultraviolet light"[All Fields]) AND (((((((((((("disinfect"[All Fields] OR "disinfectable"[All Fields]) OR "disinfectants"[Pharmacological Action]) OR "disinfectants"[MeSH Terms]) OR "disinfectants"[All Fields]) OR "disinfectant"[All Fields]) OR "disinfected"[All Fields]) OR "disinfecting"[All Fields]) OR "disinfection"[MeSH Terms]) OR "disinfection"[All Fields]) OR "disinfections"[All Fields]) OR "disinfective"[All Fields]) OR "disinfects"[All Fields]) | 2,454 |
| 3 | (ultraviolet light) AND (sterilization) | (((("ultraviolet rays"[MeSH Terms] OR ("ultraviolet"[All Fields] AND "rays"[All Fields])) OR "ultraviolet rays"[All Fields]) OR ("ultraviolet"[All Fields] AND "light"[All Fields])) OR "ultraviolet light"[All Fields]) AND ((((((((((((((((((((((((((("infertility"[MeSH Terms] OR "infertility"[All Fields]) OR "sterile"[All Fields]) OR "sterility"[All Fields]) OR "sterilant"[All Fields]) OR "sterilants"[All Fields]) OR "sterilely"[All Fields]) OR "steriles"[All Fields]) OR "sterilisation"[All Fields]) OR "sterilization, reproductive"[MeSH Terms]) OR ("sterilization"[All Fields] AND "reproductive"[All Fields])) OR "reproductive sterilization"[All Fields]) OR "sterilization"[All Fields]) OR "sterilization"[MeSH Terms]) OR "sterilisations"[All Fields]) OR "sterilised"[All Fields]) OR "steriliser"[All Fields]) OR "sterilisers"[All Fields]) OR "sterilising"[All Fields]) OR "sterilities"[All Fields]) OR "sterilise"[All Fields]) OR "sterilize"[All Fields]) OR "sterilizations"[All Fields]) OR "sterilized"[All Fields]) OR "sterilizer"[All Fields]) OR "sterilizers"[All Fields]) OR "sterilizes"[All Fields]) OR "sterilizing"[All Fields]) | 1,944 |
| 4 | (ultraviolet light) AND (infection) | (((("ultraviolet rays"[MeSH Terms] OR ("ultraviolet"[All Fields] AND "rays"[All Fields])) OR "ultraviolet rays"[All Fields]) OR ("ultraviolet"[All Fields] AND "light"[All Fields])) OR "ultraviolet light"[All Fields]) AND ((((((((((((((((((((("infect"[All Fields] OR "infectability"[All Fields]) OR "infectable"[All Fields]) OR "infectant"[All Fields]) OR "infectants"[All Fields]) OR "infected"[All Fields]) OR "infecteds"[All Fields]) OR "infectibility"[All Fields]) OR "infectible"[All Fields]) OR "infecting"[All Fields]) OR "infection s"[All Fields]) OR "infections"[MeSH Terms]) OR "infections"[All Fields]) OR "infection"[All Fields]) OR "infective"[All Fields]) OR "infectiveness"[All Fields]) OR "infectives"[All Fields]) OR "infectivities"[All Fields]) OR "infects"[All Fields]) OR "pathogenicity"[MeSH Subheading]) OR "pathogenicity"[All Fields]) OR "infectivity"[All Fields]) | 5,136 |
| 5 | (pulsed xenon ultraviolet) and (disinfection) | ((((((((("pulse"[MeSH Terms] OR "pulse"[All Fields]) OR "heart rate"[MeSH Terms]) OR ("heart"[All Fields] AND "rate"[All Fields])) OR "heart rate"[All Fields]) OR "pulses"[All Fields]) OR "pulse s"[All Fields]) OR "pulsed"[All Fields]) OR "pulsing"[All Fields]) AND ((("xenon"[MeSH Terms] OR "xenon"[All Fields]) OR "xenon s"[All Fields]) OR "xenons"[All Fields]) AND ("ultraviolet"[All Fields] OR "ultraviolets"[All Fields])) AND (((((((((((("disinfect"[All Fields] OR "disinfectable"[All Fields]) OR "disinfectants"[Pharmacological Action]) OR "disinfectants"[MeSH Terms]) OR "disinfectants"[All Fields]) OR "disinfectant"[All Fields]) OR "disinfected"[All Fields]) OR "disinfecting"[All Fields]) OR "disinfection"[MeSH Terms]) OR "disinfection"[All Fields]) OR "disinfections"[All Fields]) OR "disinfective"[All Fields]) OR "disinfects"[All Fields]) | 44 |
| 6 | (pulsed xenon) AND (hospital) | ((((((((("pulse"[MeSH Terms] OR "pulse"[All Fields]) OR "heart rate"[MeSH Terms]) OR ("heart"[All Fields] AND "rate"[All Fields])) OR "heart rate"[All Fields]) OR "pulses"[All Fields]) OR "pulse s"[All Fields]) OR "pulsed"[All Fields]) OR "pulsing"[All Fields]) AND ((("xenon"[MeSH Terms] OR "xenon"[All Fields]) OR "xenon s"[All Fields]) OR "xenons"[All Fields])) AND (((((((((((((("hospital s"[All Fields] OR "hospitalisation"[All Fields]) OR "hospitalization"[MeSH Terms]) OR "hospitalization"[All Fields]) OR "hospitalised"[All Fields]) OR "hospitalising"[All Fields]) OR "hospitality"[All Fields]) OR "hospitalisations"[All Fields]) OR "hospitalizations"[All Fields]) OR "hospitalize"[All Fields]) OR "hospitalized"[All Fields]) OR "hospitalizing"[All Fields]) OR "hospitals"[MeSH Terms]) OR "hospitals"[All Fields]) OR "hospital"[All Fields]) | 140 |
| 7 | (pulsed xenon) AND (infection) | ((((((((("pulse"[MeSH Terms] OR "pulse"[All Fields]) OR "heart rate"[MeSH Terms]) OR ("heart"[All Fields] AND "rate"[All Fields])) OR "heart rate"[All Fields]) OR "pulses"[All Fields]) OR "pulse s"[All Fields]) OR "pulsed"[All Fields]) OR "pulsing"[All Fields]) AND ((("xenon"[MeSH Terms] OR "xenon"[All Fields]) OR "xenon s"[All Fields]) OR "xenons"[All Fields])) AND ((((((((((((((((((((("infect"[All Fields] OR "infectability"[All Fields]) OR "infectable"[All Fields]) OR "infectant"[All Fields]) OR "infectants"[All Fields]) OR "infected"[All Fields]) OR "infecteds"[All Fields]) OR "infectibility"[All Fields]) OR "infectible"[All Fields]) OR "infecting"[All Fields]) OR "infection s"[All Fields]) OR "infections"[MeSH Terms]) OR "infections"[All Fields]) OR "infection"[All Fields]) OR "infective"[All Fields]) OR "infectiveness"[All Fields]) OR "infectives"[All Fields]) OR "infectivities"[All Fields]) OR "infects"[All Fields]) OR "pathogenicity"[MeSH Subheading]) OR "pathogenicity"[All Fields]) OR "infectivity"[All Fields]) | 49 |
